# Tobacco Company Efforts to Influence the Food and Drug Administration-Commissioned Institute of Medicine Report *Clearing the Smoke:* An Analysis of Documents Released through Litigation

**DOI:** 10.1371/journal.pmed.1001450

**Published:** 2013-05-28

**Authors:** Crystal E. Tan, Thomas Kyriss, Stanton A. Glantz

**Affiliations:** 1 Center for Tobacco Control Research and Education, Department of Medicine, University of California, San Francisco, San Francisco, California, United States of America; 2 Schillerhoehe Hospital, Thoracic Surgery, Gerlingen, Germany; San Diego State University, United States of America

## Abstract

**Background:**

Spurred by the creation of potential modified risk tobacco products, the US Food and Drug Administration (FDA) commissioned the Institute of Medicine (IOM) to assess the science base for tobacco “harm reduction,” leading to the 2001 IOM report *Clearing the Smoke*. The objective of this study was to determine how the tobacco industry organized to try to influence the IOM committee that prepared the report.

**Methods and Findings:**

We analyzed previously secret tobacco industry documents in the University of California, San Francisco Legacy Tobacco Documents Library, and IOM public access files. (A limitation of this method includes the fact that the tobacco companies have withheld some possibly relevant documents.) Tobacco companies considered the IOM report to have high-stakes regulatory implications. They developed and implemented strategies with consulting and legal firms to access the IOM proceedings. When the IOM study staff invited the companies to provide information on exposure and disease markers, clinical trial design for safety and efficacy, and implications for initiation and cessation, tobacco company lawyers, consultants, and in-house regulatory staff shaped presentations from company scientists. Although the available evidence does not permit drawing cause-and-effect conclusions, and the IOM may have come to the same conclusions without the influence of the tobacco industry, the companies were pleased with the final report, particularly the recommendations for a tiered claims system (with separate tiers for exposure and risk, which they believed would ease the process of qualifying for a claim) and license to sell products comparable to existing conventional cigarettes (“substantial equivalence”) without prior regulatory approval. Some principles from the IOM report, including elements of the substantial equivalence recommendation, appear in the 2009 Family Smoking Prevention and Tobacco Control Act.

**Conclusions:**

Tobacco companies strategically interacted with the IOM to win several favored scientific and regulatory recommendations.

*Please see later in the article for the Editors' Summary*

## Abstract 2

Stanton Glantz and colleagues investigate efforts by tobacco companies to influence *Clearing the Smoke*, a 2001 Institute of Medicine report on harm reduction tobacco products.

*Please see later in the article for the Editors' Summary*

## Editors' Summary

**Background:**

Up to half of tobacco users will die of cancer, lung disease, heart disease, stroke, or another tobacco-related disease. Cigarettes and other tobacco products cause disease because they expose their users to nicotine and numerous other toxic chemicals. Tobacco companies have been working to develop a “safe” cigarette for more than half a century. Initially, their attention focused on cigarettes that produced lower tar and nicotine yields in machine-smoking tests. These products were perceived as “safer” products by the public and scientists for many years, but it is now known that the use of low-yield cigarettes can actually expose smokers to higher levels of toxins than standard cigarettes. More recently, the tobacco companies have developed other products (for example, products that heat aerosols of nicotine, rather than burning the tobacco) that claim to reduce harm and the risk of tobacco-related disease, but they can only market these modified risk tobacco products in the US after obtaining Food and Drug Administration (FDA) approval. In 1999, the FDA commissioned the US Institute of Medicine (IOM, an influential source of independent expert advice on medical issues) to assess the science base for tobacco “harm reduction.” In 2001, the IOM published its report *Clearing the Smoke: Assessing the Science Base for Tobacco Harm and Reduction*, which, although controversial, set the tone for the development and regulation of tobacco products in the US, particularly those claiming to be less dangerous, in subsequent years.

**Why Was This Study Done?:**

Tobacco companies have a long history of working to shape scientific discussions and agendas. For example, they have produced research results designed to “create controversy” about the dangers of smoking and secondhand smoke. In this study, the researchers investigate how tobacco companies organized to try to influence the IOM committee that prepared the *Clearing the Smoke* report on modified risk tobacco products by analyzing tobacco industry and IOM documents.

**What Did the Researchers Do and Find?:**

The researchers searched the Legacy Tobacco Documents Library (a collection of internal tobacco industry documents released as a result of US litigation cases) for documents outlining how tobacco companies tried to influence the IOM Committee to Assess the Science Base for Tobacco Harm Reduction and created a timeline of events from the 1,000 or so documents they retrieved. They confirmed and supplemented this timeline using information in 80 files that detailed written interactions between the tobacco companies and the IOM committee, which they obtained through a public records access request. Analysis of these documents indicates that the tobacco companies considered the IOM report to have important regulatory implications, that they developed and implemented strategies with consulting and legal firms to access the IOM proceedings, and that tobacco company lawyers, consultants, and regulatory staff shaped presentations to the IOM committee by company scientists on various aspects of tobacco harm reduction products. The analysis also shows that tobacco companies were pleased with the final report, particularly its recommendation that tobacco products can be marketed with exposure or risk reduction claims provided the products substantially reduce exposure and provided the behavioral and health consequences of these products are determined in post-marketing surveillance and epidemiological studies (“tiered testing”) and its recommendation that, provided no claim of reduced exposure or risk is made, new products comparable to existing conventional cigarettes (“substantial equivalence”) can be marketed without prior regulatory approval.

**What Do These Findings Mean?:**

These findings suggest that tobacco companies used their legal and regulatory staff to access the IOM committee that advised the FDA on modified risk tobacco products and that they used this access to deliver specific, carefully formulated messages designed to serve their business interests. Although these findings provide no evidence that the efforts of tobacco companies influenced the IOM committee in any way, they show that the companies were satisfied with the final IOM report and its recommendations, some of which have policy implications that continue to reverberate today. The researchers therefore call for the FDA and other regulatory bodies to remember that they are dealing with companies with a long history of intentionally misleading the public when assessing the information presented by tobacco companies as part of the regulatory process and to actively protect their public-health policies from the commercial interests of the tobacco industry.

**Additional Information:**

Please access these Web sites via the online version of this summary at http://dx.doi.org/10.1371/journal.pmed.1001450.

- This study is further discussed in a *PLOS Medicine* Perspective by Thomas Novotny
- The World Health Organization provides information about the dangers of tobacco (in several languages); for information about the tobacco industry's influence on policy, see the 2009 World Health Organization report Tobacco interference with tobacco control
- A *PLOS Medicine* Research Article by Heide Weishaar and colleagues describes tobacco company efforts to undermine the Framework Convention on Tobacco Control, an international instrument for tobacco control
- Wikipedia has a page on tobacco harm reduction (note: Wikipedia is a free online encyclopedia that anyone can edit; available in several languages)
- The IOM report Clearing the Smoke: Assessing the Science Base for Tobacco Harm Reduction is available to read online
- The Legacy Tobacco Documents Library is a public, searchable database of tobacco company internal documents detailing their advertising, manufacturing, marketing, sales, and scientific activities
- The University of California, San Francisco Center for Tobacco Control Research and Education is the focal point for University of California, San Francisco (UCSF) scientists in disciplines ranging from the molecular biology of nicotine addiction through political science who combine their efforts to eradicate the use of tobacco and tobacco-induced cancer and other diseases worldwide
- SmokeFree, a website provided by the UK National Health Service, offers advice on quitting smoking and includes personal stories from people who have stopped smoking
- Smokefree.gov, from the US National Cancer Institute, offers online tools and resources to help people quit smoking

## Introduction

Because cigarettes and other tobacco products deliver a wide range of toxic chemicals along with nicotine, the addictive drug in tobacco, the cigarette companies mounted efforts dating back to at least the 1950s [1] to develop a “safe” cigarette. These efforts waned in the 1980s as a result of technical failures combined with industry lawyers' concerns that success in creating a “safer” cigarette would create liabilities for other “less safe” brands.

One idea that attracted wide public and scientific acceptance beginning in the 1960s was the idea that cigarettes that produced lower tar and nicotine yields (based on machine-smoking tests) would be less toxic. This assumption led to their widespread use but ultimately provided no benefit to the public health [2],[3],[4],[5]. Health authorities later learned that machine smoking tests translate poorly to how smokers actually smoke and the dose of toxins they receive. Low-yield cigarettes performed better in machine tests because cigarette companies modified the filters with ventilation holes to dilute the smoke with air, lowering the machine-measured yields. In contrast, smokers covered these holes with their fingers or lips and inhaled the unvented smoke or smoked more intensively, a process that the industry called “smoker compensation,” to maintain or even increase nicotine delivery and exposure to smoke toxins. For many years, low-yield cigarettes were perceived as “safer” products, and the tobacco industry used direct marketing claims and later, implied claims, to encourage people to initiate smoking or delay quitting [1],[2],[4].

The issue of new tobacco products that were designed to deliver nicotine accompanied with lower levels of other toxins reemerged as a public health issue in 1988 when R.J. Reynolds Tobacco (RJR) introduced its new “Premier” product, which worked by delivering a heated aerosol of nicotine rather than by burning tobacco [6].

The question of how to assess the relative harm of different tobacco products gained further currency in 1996, when the US Food and Drug Administration (FDA) asserted jurisdiction over tobacco products. To inform its regulatory efforts in 1999 the FDA commissioned the Institute of Medicine (IOM) to formulate scientific methods and standards to assess tobacco products that could potentially reduce exposure to toxicants. The IOM is part of the National Academies (which consists of the National Academy of Sciences, The National Academy of Engineering, the Institute of Medicine, and the National Research Council), elite self-selected professional organizations whose purpose is to provide scholarly advice to policy makers. Originally chartered by Congress in 1863 as the National Academy of Science, the National Academies describe their role as “produc[ing] groundbreaking reports that have helped shape sound policies, inform public opinion, and advance the pursuit of science, engineering, and medicine” [7]. The National Academies describe their reports as influential because, “Over many decades, the National Academy of Sciences (NAS), National Academy of Engineering (NAE), Institute of Medicine (IOM), and National Research Council have earned a solid reputation as the nation's premier source of independent, expert advice on scientific, engineering, and medical issues” [8]. Thus, while not a regulatory body per se, the IOM's advice carries much weight with policy makers and regulatory bodies, including the FDA.

FDA's charge to the IOM was to consider four questions about harm-reduction tobacco products in general [3]:

1 **Does use of the product decrease exposure to the harmful substances in tobacco?**
2 **Is this decreased exposure associated with decreased harm to health?**
3 **Are there surrogate indicators of this effect on health that could be measured in a time frame sufficient for product evaluation?**
4 **What are the public health implications of tobacco harm-reduction products?**

In response, the IOM formed its Committee to Assess the Science Base for Tobacco Harm Reduction, composed of 12 experts in fields ranging from toxicology to epidemiology from inside and outside tobacco control [3], supported by IOM staff, nonvoting liaisons from other IOM boards, and nonvoting consultants. The committee gathered information from scientists and advocates representing public health and academia and compiled a draft report, which was submitted to peer reviewers selected by the study staff before being released to the public.

The final report, *Clearing the Smoke: Assessing the Science Base for Tobacco Harm Reduction* [3] was issued in 2001 and set the tone for future development and regulation of tobacco products, particularly products claiming to be less dangerous than conventional cigarettes. The report, which included a set of Regulatory Principles (Box 1) in addition to a review of the science base, generated controversy within the tobacco control community because some believed the recommendations did not adequately protect public health [9].

**Box 1. IOM *Clearing the Smoke* Regulatory Principles [3]**

1. Manufacturers of tobacco products, whether conventional or modified, should be required to obtain quantitative analytical data on the ingredients of each of their products and to disclose such information to the regulatory agency.
2. All tobacco products should be assessed for yields of nicotine and other tobacco toxicants according to a method that reflects actual circumstances of human consumption; when necessary to support claims, human exposure to various tobacco smoke constituents should be assessed using appropriate biomarkers. Accurate information regarding yield range and human exposure should be communicated to consumers in terms that are understandable and not misleading.
3. Manufacturers of all PREPs should be required to conduct appropriate toxicological testing in preclinical laboratory and animal models as well as appropriate clinical testing in humans to support the health-related claims associated with each product and to disclose the results of such testing to the regulatory agency.
4. Manufacturers should be permitted to market tobacco related products with exposure-reduction or risk-reduction claims only after prior agency approval based on scientific evidence (a) that the product substantially reduces exposure to one or more tobacco toxicants and (b) if a risk reduction claim is made, that the product can reasonably be expected to reduce the risk of one or more specific diseases or other adverse health effects, as compared with whatever benchmark product the agency requires to be stated in the labeling. The “substantial reduction” in exposure should be sufficiently large that measurable reduction in morbidity and/or mortality (in subsequent clinical or epidemiological studies) would be anticipated, as judged by independent scientific experts.
5. The labeling, advertising, and promotion of all tobacco related products with exposure-reduction or risk-reduction claims must be carefully regulated under a “not false or misleading” standard with the burden of proof on the manufacturer, not the government. The agency should have the authority and resources to conduct its own surveys of consumer perceptions relating to these claims.
6. The regulatory agency should be empowered to require manufacturers of all products marketed with claims of reduced risk of tobacco-related disease to conduct post-marketing surveillance and epidemiological studies as necessary to determine the short-term behavioral and long-term health consequences of using their products and to permit continuing review of the accuracy of their claims.
7. In the absence of any claim of reduced exposure or reduced risk, manufacturers of tobacco products should be permitted to market new products or modify existing products without prior approval of the regulatory agency after informing the agency of the composition of the product and certifying that the product could not reasonably be expected to increase the risk of cancer, heart disease, pulmonary disease, adverse reproductive effects or other adverse health effects, compared to similar conventional tobacco products, as judged on the basis of the most current toxicological and epidemiological information.
8. All added ingredients in tobacco products, including those already on the market, should be reported to the agency and subject to a comprehensive toxicological review.
9. The regulatory agency should be empowered to set performance standards (e.g., maximum levels of contaminants; definitions of terms such as “low tar”) for all tobacco products, whether conventional or modified, or for classes of products.
10. The regulatory agency should have enforcement powers commensurate with its mission, including power to issue subpoenas.
11. Exposure reduction and risk reduction claims for drugs that are supported by appropriate scientific and clinical evidence should be allowed by the FDA.

The tobacco companies have a long history of working to shape scientific discussions and agendas [1],[4],[5], including producing research results designed to “create controversy” about the dangers of smoking and secondhand smoke [1],[10],[11],[12] and influencing scientific standards of how research is conducted or interpreted [13],[14],[15]. The cigarette companies used this experience as the basis for their efforts to influence the IOM. They worked with consultants and lawyers to gain access and involvement with the IOM process and to contribute scientific information to the IOM committee that was largely produced by industry insiders and consultants and carefully vetted by lawyers. While available evidence does not permit cause-and-effect conclusions, and the IOM may have come to the same conclusions without the influence of the tobacco industry, in the end, the companies were pleased with the report and sought ways to use it to advance their business and regulatory agendas.

## Methods

Between May 2011 and February 2012, we searched the UCSF Legacy Tobacco Documents Library (LTDL, http://legacy.library.ucsf.edu) for documents outlining how the tobacco companies tried to influence the IOM committee. We used standard snowball techniques [16], starting with terms including “Institute of Medicine,” “IOM committee,” “Clearing the Smoke,” and “Assessing the Science Base for Tobacco Harm Reduction.” We identified key persons, concepts, and events and viewed documents with adjacent Bates numbers.

Initial keyword and snowball searches yielded several thousand documents pertaining to the IOM project. Searches were narrowed to documents from specific years, namely 1999 and beyond. For documents that were exact duplicates, only one copy was included in the analysis. If the same document appeared with minor changes (i.e., handwritten notes or tracked changes in a word editing program), both versions were included.

The tobacco industry documents were analyzed for relevance, novel information, and internal consistency. Documents that were irrelevant (i.e., misclassified with metadata attributed to the wrong year, author, or topic) were excluded from the analysis. Documents that did not provide novel information (i.e., providing information that was already corroborated by other documents) were considered in the analysis for internal consistency (see below) but may not have been quoted in the final paper and do not appear in the reference list.

The remaining tobacco industry documents (about 1,000 documents) were analyzed for internal consistency. This was conducted by placing the documents in chronological order and quoting or summarizing each document to create an extensive timeline of events. The timeline was verified for content and internal consistency by authors CET and SAG. In cases where content of documents was ambiguous, additional searches for related documents were run to provide context and clarify meaning; if no supporting documents could be identified, the document was excluded from the analysis. No major conflicts arose within the documents or between the documents and known events.

In addition, documents were obtained via a public records access request to the NAS for documents associated with the IOM Committee to Assess the Science Base for Tobacco Harm Reduction. The Public Access Records Office (PARO) provided a master list of 268 documents meeting these criteria. We requested all 268 files; PARO provided 16 hard-copy documents and 64 digital files. The remaining items were papers, books, government documents, and media reports that were publicly available and not explicitly generated for or by the IOM committee. The 80 files we obtained contained all known written interactions between the tobacco companies and IOM. The timeline of events developed from the tobacco industry documents and supplemented and confirmed by the IOM documents naturally coalesced into thematically united sections; that is, documents from a particular period tended to address the same topic. Examples include: information gathering about IOM, lawyers editing presentations of industry scientists, and executives discussing the report after release. The final paper was written based on these thematically united segments.

## Results

### Phillip Morris Engages the IOM Committee

In 1999, when the FDA commissioned the IOM to prepare the report, the tobacco industry and FDA were engaged in a protracted legal contest over authority to regulate tobacco products ([3], p. 127–128; [17]). Despite their outward resistance, tobacco companies were internally acknowledging and preparing for regulation [17],[18]. A 1999 internal presentation at Philip Morris (PM) titled “Potential for Worldwide Product Regulation” described not only the IOM committee but also tracked the progress of several other tobacco regulatory efforts in Canada, the European Union, and the World Health Organization (WHO). This presentation identified harm reduction as a “critical regulatory issue” and suggested an approach by which, “in situations where significant questions remain, such as identifying important smoke components, obtaining meaningful exposure information and developing measures of harm reduction, it would be *desirable to work with regulators* to jointly address and answer these questions [emphasis added]” [18].

In October 1999, the IOM announced the creation and goals of the committee [19]. PM recognized an opportunity to influence the scientific evaluation and regulation of reduced-harm products. Through its Worldwide Regulatory Affairs (WRA) and Worldwide Scientific Affairs (WSA) divisions, PM began working with lawyers and consultants to collect information and plan ways to become involved. Established in 1993, WRA was a central resource to develop strategies and provide resources for PM Corporate Affairs staff to address smoking restrictions globally. WRA guided WSA to “develop scientific resources and contributions to the scientific debate” [20], initially on secondhand smoke and later on a wider range of scientific domains, including reduced-harm products.

In mid-November 1999 Arnold & Porter (A&P), a law firm representing PM, wrote Mark Berlind, Senior Assistant General Counsel of WRA, and Rick Solana and Bruce Davies, respectively Vice President and Manager of WSA, describing a telephone conversation A&P had had with Dr. Kathleen Stratton, the IOM study director. A&P reported that Stratton explained the committee structure, funding source (the FDA), staff, membership, and schedule [21]. A&P also gathered information about the degree to which industry representatives could be involved at every stage; A&P's memo to PM indicated that although IOM committees typically did not permit industry participation given potential conflict of interest situations, Stratton had anticipated that industry (both pharmaceutical and tobacco companies) would be encouraged to present information to the committee, either through testimony or submissions (including reference to relevant documents). A&P also indicated that, because peer reviewers on the draft committee are not subject to conflict of interest rules, it would be possible for members of industry to serve as peer reviewers. (The IOM used a lawyer from the Covington & Burling law firm, which represents the tobacco industry, as a peer reviewer.)

PM hired Multinational Business Services, Inc. (MBS), a lobbying and consulting firm founded by former Reagan White House Office of Management and Budget deputy administrator Jim Tozzi that specializes in regulatory issues and has a long history of working for the tobacco industry [13],[14] to provide options for PM to “enter the IOM process” [22]. To overcome the IOM's closely guarded decision-making process, MBS suggested two ways PM could approach the IOM:

**Option 1: Efforts could be made to work with a nationally renown [*sic*] scientific organization to establish a panel which could undertake a course of inquiry parallel to that of the IOM committee. This new panel would work in concert with Philip Morris scientists in conducting its research. At a suitable time, the panel, in the course of its interactions with the IOM committee, could bring Philip Morris officials into the dialogue.**
**Option 2: Philip Morris and MBS could jointly approach the IOM Committee through our established contacts. [22]**

PM's Solana began coordinating efforts to contact the IOM committee along with other members of WSA who would become key players: Richard Carchman, vice president of WSA; Wolf Reininghaus, head of Institut für Biologische Forschung (INBIFO, PM's biological research lab in Germany [23], renamed Philip Morris Research Laboratories GmbH in 2002 [24]); PM principal scientist George Patskan; PM scientific affairs manager Bruce Davies; and WSA group director Edward Sanders. Their initial plan was to critique the committee's “limited” range of expertise [25],[26],[27],[28],[29]. Davies recommended additional or alternate committee members, all of whom were affiliated with the tobacco industry: Bill (William) Rickert, who had been chair and editor of the 1996 and 1998 reports of Canada's Expert Committees on Cigarette Modifications and Cigarette Toxicity Reduction [30],[31] and owner of Labstat Corporation, which within the next year would sign a two-year US$950,000 contract with PM “to provide services relating to the testing and chemical analysis of tobacco, cigarettes, and cigarette smoke for constituents of interest” [32]; PM's Richard Carchman; and Oak Ridge National Laboratory's Roger Jenkins, a scientist who had a history of producing research that supported the industry's positions, particularly on secondhand smoke [33],[34].

Shortly after WSA's discussion of the IOM committee, MBS's Tozzi wrote PM that “the next step would be to raise the possibility of … data sharing with the IOM Committee” [35]. Using the information and recommendations from A&P and MBS, Solana and his WSA colleagues addressed a letter to the IOM committee on behalf of PM:

**You have posted invitation for public comment on the committee for “Assessing the Science Base for Tobacco Harm Reduction” …**
**…[I]t is not clear that you have scientists with an in depth knowledge of cigarettes and cigarette smoke. I understand that industry scientists are not allowed to be on the committee. We are, therefore, available to you to share our knowledge, experience and expertise in product design and product performance, biological and chemical evaluation of cigarettes, and capability of different tests for use in toxicological evaluation. Attached is a reference list of some of our applicable publications and presentations. [36]**

Solana's letter also followed MBS's advice [22] to cite the example of Canada's Expert Committees on Cigarette Modifications (1996) and Cigarette Toxicity Reduction (1998), which were Canada's attempts to establish priorities in tobacco harm reduction [36]. The Canadian committees had a strong tobacco industry presence; they included industry scientists J. Donald Bethizy (RJR), Patrick Dunn (Imperial Tobacco), and David Townsend (RJR) on the 1996 10-member committee; Bethizy and Hoffman again as well as Richard Carchman (PM) and Stewart Massey (British American Tobacco, Imperial Tobacco) on the 1998 11-member committee; non-disclosed industry consultant Roger Jenkins; and chair Bill Rickert. The Canadian reports included pro-industry minority opinions in the main report, immediately underneath the majority conclusions [30],[31]. These minority opinions from the industry representatives challenged widely accepted facts, including what Rickert described in his preface as committee chair as “major disagreement” about whether nicotine was addictive [30].

MBS and A&P attended the first IOM committee meeting (open to the public) in December 1999 and provided PM with detailed reports on the charge to the committee, the questions asked by the committee members, and their reactions to the answers. A&P also prepared detailed personal reports on all the committee members that included publications and sponsors [37],[38], which PM supplemented with resumes [39],[40],[41],[42],[43],[44],[45],[46],[47],[48],[49],[50],[51],[52],[53],[54],[55],[56],[57],[58],[59].

Shortly after the first committee meeting, IOM study director Stratton responded to Solana's request stating:

**I am currently working with some of the committee members to develop a strategy for engaging the pharmaceutical and tobacco industries in order to broaden the input to and the scientific base of our committee's deliberations. I am happy to know of your personal interest in our work and will take the liberty of contacting you directly once this working group plans its next steps. [emphasis added by PM] [60].**

Solana forwarded the email to several PM scientists who would play key roles in the company's upcoming interactions with the IOM: Bruce Davies, Hans-Juergen Haussman, George Patskan, Wolf Reininghaus, Edward Sanders, and Roger Walk, as well as Jack Nelson, PM senior VP of Operations [61]. Though only Solana communicated directly with Stratton, he shared their communications with many PM scientists, executives, and lawyers who worked together to collectively formulate his responses.

### IOM Invites Tobacco Companies to Share Information

In January 2000, Stratton sent identical letters to the scientific division leaders at PM [62], RJR [63], and Brown & Williamson (B&W) [64] inviting participation in an IOM meeting. The invitation advised that an IOM working group wished to “explore how you can best provide meaningful scientific information for the committee's consideration” and asked the companies for scientific articles and company documents made public under the Master Settlement Agreement or by congressional action. The letter further noted, “…The invitation to meet with the working group and solicitation of input is not an endorsement of the products of your company or positions you might take regarding the health effects of tobacco or nicotine” [62],[63],[64].

Solana responded to Stratton's request for materials and references [65] and sent several copies of *Analytical Determination of Nicotine and Related Compounds and Their Metabolites* [66], a 772-page monograph on nicotine analysis PM commissioned, sponsored, and primarily written by industry scientists, consultants, and grantees [67]. Though the monograph stated that PM paid some of the cost of the book and that some contributors were employees, affiliates, or consultants of tobacco companies, it did not disclose that PM scientists, lawyers, and management at PM and RJR actively revised chapters or that PM agreed to purchase a minimum of 500 copies to make production of the monograph profitable for the publisher [67].

PM selected six speakers to present to the IOM: Richard A. Carchman (VP of WSA), Hans-Juergen Haussman (Executive Manager, Bioresearch at INBIFO), George Patskan (Director of Product Integrity), Richard Solana, and Principal Scientists from both PM and PM International [68]. Their presentations were carefully vetted by the “Core Team” of WSA and INBIFO research scientists [69] as well as in-house lawyers (Senior Vice President and Associate General Counsel and Vice President of Litigation and Associate General Counsel) [70]. Another PM lawyer, Kevin Osborne, helped edit the presentations [71],[72],[73]. A list of scheduled planning sessions for the presentations also indicated that a 4-hour session would involve a review by PM Vice President of Operations Jack Nelson [69]. Thus, while the primary work was conducted by the science personnel, they operated under the guidance of PM's legal, regulatory, and executive arms.

Sanders presented research by British statistician Peter Lee on low tar cigarettes and argued that “direct epidemiological evidence suggest[s] some reduction in risk for lung cancer” while “indirect epidemiological evidence appears to suggest the reverse” and that this “require[s] further research” [74]. Lee, a longtime tobacco industry consultant, had written articles denying or minimizing the health effects of cigarette smoking on behalf of British American Tobacco, PM, the Tobacco Institute, and others ([4], p. 86; [1],[10],[75],[76],[77],[78]), and in this case wrote an article concluding that “the switch to low tar/filter cigarettes has led to a substantial reduction in risk of lung cancer” [79]. This statement directly contradicted the scientific consensus, developed over several decades, that low-yield products failed to reduce population-wide risks of smoking [2].

When the IOM working group asked PM for a copy of Lee's study, which PM stated had been submitted to *British Medical Journal* [80], PM did not comply. Sanders informed the PM staff member fulfilling the IOM data request that, “The reason for [the refusal] is, of course, that if the article is rejected, it may be changed considerably” [81]. *BMJ* subsequently rejected the manuscript, and it was eventually published [82],[83] in 2001 in *Inhalation Toxicology*, a journal whose editor-in-chief [84] was a paid RJR consultant [85],[86],[87],[88],[89],[90], after receiving a positive review by peer reviewer Chris Coggins, Senior VP of Science and Technology at RJR, who declared it a “fine piece of epidemiological research [that] *is suitable for publication with very minor changes* [emphasis in original]” [91].

Another presentation to the IOM was by PM Principal Scientist Patskan who delivered a presentation about the smoke chemistry and toxicity of electronically heated cigarettes, new devices that claimed to reduce the risk of smoking by heating rather than burning tobacco. Patskan requested that his research colleagues send him results from assays and inhalation studies on a “TPM [total particulate matter] delivery basis” [92]. Previously, in its “Project Mix,” PM used the tactic of normalizing smoke yields by TPM delivery in order to obscure increases in cigarette smoke toxicity that occurred when additives were put in test cigarettes [93].

Like PM, RJR delivered high-priority talking points to the IOM, one of which was that modified products must be as similar to conventional cigarettes as possible, because the “degree of trade-off strongly influences cigarette acceptability” [94]. RJR cited their own data collected from smokers participating in company-run trials of Eclipse, their then-new tobacco-heating “cigarette” that advertised simpler smoke chemistry and reduced biological activity. The data showed that smokers were “unwilling to accept large trade-offs” of the taste, ritual, or performance for a risk reduction, causing RJR to recommend that regulators not require drastic modifications of tobacco products that would make them unattractive to consumers [94].

RJR also presented data showing that Eclipse produced less tar and other target compounds, such as carcinogenic polyaromatic hydrocarbons and tobacco-specific nitrosamines, than conventional low-yield cigarettes according to machine smoking and smoke composition tests [94]. After RJR's presentation to the IOM, the company's public relations department drafted a briefing sheet for media training about Eclipse (also sent to law firm Williams & Connolly to request suggested changes [95]) stating, “We have presented the science behind our claims to … the Institute of Medicine's committee on cigarette risk reduction, and to others in the scientific and public health communities” [96].

PM, RJR, and B&W all recommended similar test batteries for smoke chemistry and toxicology tests (the Ames test, neutral red uptake assay, sister chromatid exchange, chromosome aberration assay, 90-day subchronic inhalation studies in mice, and skin painting tests) as well as similar biomarkers for disease outcomes such as cardiovascular disease, chronic obstructive pulmonary disease, and cancer [94],[97],[98].

Following the presentations to the IOM working group, PM's Solana circulated an email to WSA [99] reporting on the meeting and expressing pleasure that “there was no animosity, and the working group was sincerely interested in our information and thoughts” [100], and that the committee inquired about the company's research collaborations, capabilities, and agenda as well as their opinion on the IOM's work and a regulatory framework for reduced-harm products. He described the “key messages” conveyed by PM, which included “us[ing] a balanced spectrum of assays for pre-market hazard characterization” such as machine smoking, “a useful tool [that] should use a validated, standard method,” and “confirm[ing] harm reduction determination with after-market epidemiology.” Solana reported that he told the working group that “PM will be glad to provide support to, and receive support from, this IOM committee” [101]. Finally, Solana mentioned that the IOM committee would be sending a list of further questions to be answered by the companies.

### IOM Sends Twelve Questions to the Tobacco Companies

Six weeks after the company presentations, the IOM sent 12 questions on tobacco products and testing standards to PM, RJR, B&W, and Lorillard (Table 1) [102],[103],[104],[105]. Their responses were collaborative efforts within each company, with input from scientists, lawyers, and regulatory advisors to create carefully crafted responses [106],[107],[108],[109].

**Table 1:** IOM's Twelve Questions to the Tobacco Companies, Their Responses, and the Conclusions in *Clearing the Smoke*.

IOM Question	Excerpts from Tobacco Company Responses [94],[97],[98]	IOM Final Conclusion
1. What evidence is there to conclude that there is or is not a threshold effect for cancer, heart disease, adverse reproductive effects or lung disease caused by exposure to tobacco or tobacco smoke, and what is the threshold level, if it exists[?]	*PM*: “There is no generally agreed safe level of smoking based on the epidemiological data available to date. However, conceptually, thresholds are conceivable but may not be seen due to the complexity of the exposure and the disease processes as well as a lack of resolution of the epidemiological dosimeter […] Conceptually, thresholds most likely exist for a number of mechanistic events in smoking-related diseases.*RJR*: “It is very difficult to prove experimentally that a threshold exists or does not exist, since the difficulties of scientifically proving a negative are well known. Nevertheless, based on mechanistic considerations such as DNA repair, detoxification, and other biological defense mechanisms for preserving homeostasis, it is widely accepted that a threshold does exist for most, if not all, chemical agents.”	“[T]here is currently no evidence to support a threshold level of tobacco exposure below which no risk exists for any of the reviewed health outcomes.” (p. 9)
2. Do the filters in your cigarettes, including reduced-risk products under test-market or development, contain fibers? If so, can these fibers become airborne in the smoke inhaled by smokers? If so, to what extent does this occur? What is your estimate of the potential risk to human health from these fibers?	*PM*: “The potential human health risk from exposure to cellulose acetate fibers is low since the probability of deposition in the lower respiratory tract is very low or zero. This conclusion is based upon expert opinions expressed in the scientific literature and experimental data […] Currently, there are no scientific studies published in the literature that provide definitive evidence that cellulose acetate fibers from cigarette filters can penetrate to the deep lung in the human.*RJR*: “Although the question posed by the Committee refers to fibers within *filters*, R.J. Reynolds assumes that the Committee's actual interest relates to the continuous filament glass composing the glass mat insulator of Eclipse due to some published speculation about those fibers… [emphasis in original]”	“This report has not reviewed potential cancer risks due to fibers released from cigarette filters or tobacco additives, **because it is thought that the risk of these exposures are substantially lower than the risk from the constituents of tobacco smoke.** However, there are no existing data to prove this assumption.” (p. 165)
3. What is the effect of removing a component or components of tobacco on the qualitative and quantitative composition of the new species of smoke? What is the empirical evidence that such alteration would be associated with reduced or altered disease incidence in humans?	*PM*: “Removal of components has been attempted but has rarely resulted in a **commercially acceptable product**. They often resulted, as in the case of nicotine removal, in serious taste changes of the smoke of the modified cigarettes so that these products were not viable in the market place and, hence, no harm reduction was obtained. In other cases, e.g. nitrate, the significance for hazard reduction was unclear since the changes of biological responses could not be related to hazard reduction unambiguously. Most recent efforts, e.g., protein and TSNA, however, are promising.”“When 333 ingredients (for technical reasons split into three groups) were added to American-blend commercial cigarettes at an appropriate use level and at a multiple of the normal use level, an **extensive chemical analysis of the resulting smoke indicated essentially no major changes**.” (Author's note: The research in question, titled **Project MIX**, was found to have been presented in a strategically misleading way to prevent anticipated tobacco control regulations [93].)*RJR*: “Four of the key technologies RJR has explored for removing or reducing specific components of tobacco are […] deproteinization of tobacco, selective filtration, reduction of tobacco-specific nitrosamines, and tobacco-heating technology [Eclipse] […] We should also point out that our experience has been that generally any new technology, when incorporated into cigarette design, presents significant consumer acceptance issues. These issues must also be addressed if smokers are to realize the benefits of a cigarette with the potential to present less risk.”	“[T]here are insufficient data to allow scientific judgement [sic] or prediction of the health effects of removal of one class of chemicals from tobacco products.” (p. 234)
4. What are the criteria that should be used to assert that a specific form of tobacco or tobacco product is less harmful than others? What biomarkers should be used to assess the criteria?	*PM*: “The process would include a pre-market acceptability evaluation, which would involve smoke chemistry and both in vitro and in vivo toxicology testing to insure that a new product design change **does not increase overall smoke chemistry or measured biological activity**.Once the product is on the market, an exposure assessment would be conducted. Due to the need for large numbers of smokers who currently use a product as their brand, it would be best to conduct this study in an after-market environment. [emphasis added]	“Toxicology studies, both in vitro and in vivo, provide the opportunity to evaluate the potential harm reduction offered by potential reduced-exposure products (PREPs) […] The preclinical tests should include in vitro tests in both animal and human cells to determine the cytotoxicity and the genotoxicity of the tobacco product to which humans will be exposures. Such a test must include dose-response studies to determine the amount of the exposure material required to cause toxicity. Next, studies should be conducted in vivo in the best animal models available to determine the comparative potency of the PREP versus the standard product […] If these preclinical studies indicate that the PREP is less potent than the standard tobacco product, clinical studies should be conducted to determine acute toxic effects, the toxicokinetic properties, or the adverse effects of the PREP in humans. [T]he testing approach will allow the rejection of risk reduction claims for products that are as toxic or more toxic in preclinical tests compared to products already on the market; however, only after long-term use of the product by many people could it be determined if the chronic toxicity of the new product is less than that of the standard product.(p. 302–303)It is beyond the scope of the committee's task to recommend the specific set of toxicity tests that should be done on new or existing tobacco products.”(p. 303)“**Regulatory Principle 7. In the absence of any claim of reduced exposure or reduced risk, manufacturers of tobacco products should be permitted to market new products or modify existing products without prior approval of the regulatory agency after informing the agency of the composition of the product and certifying that the product could not reasonably be expected to increase the risk** of cancer, heart disease, pulmonary disease, adverse reproductive effects or other adverse health effects, compared to similar conventional tobacco products, as judged on the basis of the most current toxicological and epidemiological information.” (p. 10)“It is unclear how much actual reduction in harm should be required for approval and marketing of a harm reduction product.” (p. 54)
5. What is the appropriate comparison product for reduced-risk products? A Kentucky reference cigarette? The leading product as assessed by market share? The lowest-risk product currently available? Each individual smoker's brand at time of switching to the new product? Each individual smoker's dominant brand of his/her smoking history?	*PM*: “For studies of smoke chemistry and toxicity, the key purpose of the comparison cigarette is to provide an anchor point […] In this regard, the University of Kentucky reference cigarette 1R4F is suitable at this time.For human studies of either exposure or health effects, commercially available cigarettes would be preferred.”*RJR*: “Studies demonstrate the acceptability of the University of Kentucky 1R4F and 1R5F reference cigarettes as appropriate models for the marketplace categories of full flavor, low-tar and ultra low-tar, respectively […][Chemical testing and analysis] and [biological and toxicological testing] is best executed with standardized reference cigarettes like the University of Kentucky 1R4F and 1R5F products.[S]tudies in smokers are best accomplished by comparisons to the smoker's usual brand.”	“Depending on study design, different exposure comparisons can be made, some of which are more applicable than others. For the development of biomarkers that are tested first in the laboratory setting (in vitro and in vivo animal studies), authentically synthesized tobacco carcinogens, or components of a reference cigarette (i.e., the tar fraction or cigarette smoke condensate from a Kentucky reference cigarette), would be preferable.”(p. 438)“Experimental human studies in which the product is initially tested would optimally be compared to both reference cigarettes and separately to the smokers' usual cigarettes.” (p. 303)
6. What endpoints would you recommend for assessing harm reduction through use of reduced risk products or by decreasing consumption of existing products via use of nicotine replacement therapies or other pharmaceutical products?	*PM*: “It should be clear that the extent of testing to support a claim on harm reduction exceeds what is required in terms of acceptability testing for market introduction of changes in cigarette design or ingredient additions.**We consider four tiers of endpoints to be useful to assess the potential for harm reduction: chemical smoke analysis, experimental toxicology, clinical tests, and epidemiology. These tiers go beyond what currently is considered necessary for market acceptability testing.** Chemical smoke analysis and experimental toxicology should be performed before any test marketing. Clinical test could be performed after market introduction in order to support provisional product claims. Epidemiology is only possible long after market introduction to confirm product claims. Endpoints for chemical smoke analysis and experimental toxicology endpoints in the context of carcinogenicity are available and being routinely used, while they need to be further validated for non-neoplastic diseases. For clinical tests, there is no common understanding regarding the choice of endpoints, and a flexible and scientifically reasonable approach is needed to obtain consensus among the stakeholders. Epidemiological data are needed on the long run to ascertain the intended harm reduction and to further validate experimental and clinical testing strategies.”*RJR*: “Biomarkers of cigarette-related risk should emphasize the following: 1) cancer risk biomarkers: measurements of potential DNA alteration and increased rates of cell proliferation as suggested by cellular morphology, cell surface markers, and exposure to mutagens; 2) cardiovascular risk biomarkers: measurements of atherogenic and thrombogenic potential; 3) COPD biomarkers: indicators of pulmonary inflammation.There are […] four major problems inherent in attempting to conduct a prospective epidemiology study on a reduced risk cigarette-type product: [paraphrased] the lag phase for carcinoma can be on the order of decades (and some smokers may be neoplastically “initiated” by prior use of tobacco-burning cigarettes), a very large number of reduced risk cigarette smokers would need to be enrolled, some smokers simultaneously smoke some tobacco-burning cigarettes (non-compliance), and smokers willing to switched to a reduced risk cigarette-type product, who are willing to make a trade-off in terms of taste, sensory impact and ease of lightability may not be behaviorally or demographically comparable to the average smokers.”	Disease endpoints of interest covered in the report include: cancer, cardiovascular disease, non-neoplastic respiratory diseases, reproductive and developmental effects, and “other” health effects (e.g., gastrointestinal, rheumatologic, psychiatric, neurologic disease) (p. 367–573)
7. What clinical exposure data in humans do you feel is important or even necessary for evaluation of a new tobacco product (smoked and smokeless) before it is marketed? Please be specific with respect to number of subjects, length of use, biomarkers measured, and tests conducted.	*PM*: “**We believe that as long as a new product does not increase the hazard of smoking, it should be allowed into commercial sales**. However, before any claim of reduced harm is made, human exposure measurements are an important component of data relevant to supporting such claims. [Refer to Figure 1]The variability in the conditions under which cigarettes are naturally used and the requirements for very large populations for statistically meaningful results require that exposure measurements be conducted **post-marketing.”**	“If … preclinical studies indicate that the PREP is less potent than the standard tobacco product, clinical studies should be conducted to determine acute toxic effects, the toxicokinetic properties, or the adverse effects of the PREP in humans.”(p. 302–303)“The committee recommends that a panel of experts by convened to determine the specific set of toxicity tests and details of the testing regiments. Details to be considered include species and strains of test animals, duration of tests, end points of interest, dose-response considerations, biomarkers of dosimetry and response, and standard comparison products to be tested as positive and negative controls.” (p. 303)
8. If a goal is to develop a reduced-risk product that is **still attractive to smokers who wish to continue smoking**, what research agenda would you recommend? What do you need to know to accomplish this that you do not know already? Consider same question for smokeless tobacco products and comment on the possibility suggested by Swedish Snus that taste can be maintained while risk is minimized.	*PM*: “**A reduced harm product that is unacceptable to the consumer is of little value.** It does not advance our harm reduction efforts, does not benefit the public health community that hopes to transition inveterate smokers to a reduced harm product, and does not benefit the consumer, who stands to directly benefit from such a product. There, to be commercially viable and to accomplish the goal of providing harm reduction to that population of smokers who do not quit, any new reduced harm product must possess characteristics that smokers deem to be desirable.”*RJR*: Smoking is a very complex behavior. **Any attempt to modify the product or smokers' behavior must be acceptable to the consumer**.”	“**Retaining nicotine at pleasurable or addictive levels while reducing the more toxic components of tobacco is another general strategy for harm reduction.** The tobacco industry reportedly would support some FDA regulation of cigarette products. **Key to its acceptance is that there be no upper level for nicotine that is set so low as to effectively ban cigarettes.** Experience with NEXT, a cigarette with extremely low nicotine levels that did not succeed in the marketplace, suggests that nicotine is one of the factors crucial to the success of a tobacco product.” (p. 29)[From public statement about report by Committee Chair Stuart Bondurant] “**We believe that manufacturers should have the necessary incentive to develop and market these products. What I mean by this is that there be a regulatory framework that is not so burdensome that manufacturers are not able to get these products to market but strict enough that they do in fact qualify as harm reduction products.**”[140]
9. What is the extent of your plans for internal or external activities in supporting health outcomes studies for your products? Particularly, do you support or plan to support epidemiology or related investigations?	*PM*: Our current plans in this regard center mainly on exposure assessment […] We are currently considering, as well, the evaluation of short-term, less than one year, health endpoints in human studies. These studies would be conducted as endpoints are identified that are plausibly predictive of chronic disease**. While we will be pursuing this area of research, the limiting factors are guidance from and cooperation of the public health community, as well as regulatory guidance**. Reduced harm products, which are deemed so only by industry scientists, not communicated to the consumer, and therefore not used, will be of little value. Hence, out definitive plans will follow the lead of the public health community and regulatory bodies. That is why our efforts are focused on supporting groups such as this IOM Committee in working through these questions.”*RJR*: “At this point, out plans regarding post-market surveillance have not been finalized. Such a project presents many design and subject compliance issues which we are still working through” including anonymity of tobacco product users and limits of long-term epidemiological investigations [see RJR response to question 6].	The report does not directly address this issue.
10. What might be the best mechanisms to foster collaborative studies between tobacco industry, university, and other scientists?	*PM*: “Important ingredients for collaborative interactions between the tobacco industry, academia and government and non-government scientists are open and frequent communication, rigorous scientific peer review of scientific work, and transparent processes for both funding and scientific review. These are the tools with which trust will flourish.We are a willing participant of such interactions and bring talented research resources and expertise to such communication and collaboration. It is now Philip Morris USA's written mission statement to be the most responsible, effect, and respected developer, manufacturer, and marketer of consumer products made for adults. In doing so, we follow written company values of integrity, trust and respect, executing with quality, and sharing with others.” [PM advocated the following tools: communication, peer review, and transparent and open process.]*RJR*: “The Committee is in a unique position to foster such collaborative studies by taking an affirmative stand that no stigma be attached to university and other scientists who participate in collaborative studies with tobacco industry scientists.[…] We at RJRT would like to call the Committee's special attention to one of [the Society of Toxicology's] four principles: ‘Research should be judged on the basis of scientific merit without regard for funding source or where the studies are conducted […]’”	The report does not directly address this issue.
11. What roles should various sectors (e.g. industry, academia, government, foundations) play in the design, funding, oversight, reporting, and modification of post-marketing surveillance of newly introduced reduced risk products?	*PM*: [Acknowledging a role for industry, academia and scientific bodies, and government] “**The tobacco company should take the lead role in post-market surveillance studies**; however, the process cannot work without clear and open communication and collaboration between the stakeholders. The design, execution, modification, and reporting of post-market surveillance studies will have participation from all of the stakeholders you mention.”*RJR*: “The manufacturer of a specific newly introduced reduced risk product should bear the primary responsibility for post-marketing surveillance in the short term, although with as much collaboration from academia, government, and foundations as the manufacturer can secure.RJRT believes that reasonable and workable federal government oversight of reduced risk products is desirable […] At present, however, there is no mechanism in place for reasonable oversight of all aspects of such products […] We encourage the Committee to have a conference to gather ideas on how federal oversight might be most effectively inaugurated.”	**Regulatory Principle 6.** [A] regulatory agency should be empowered to require manufacturers of all products marketed with claims of reduced risk of tobacco-related disease to conduct post-marketing surveillance and epidemiological studies as necessary to determine the short-term behavioral and long-term health consequences of using their products and to permit continuing review of the accuracy of their claims. (p. 10)
12.What is your thinking on the design of a population-based study to assess the effects of the introduction of a reduced-risk product on risk perception of tobacco use and on tobacco initiation, cessation, or relapse?	*PM*: “While a goal in the efforts to protect public health would seem to be to reduce the number of people utilizing hazardous products, the ultimate goal would be to decrease the population risk. **Long-term post-market surveillance studies would evaluate the accomplishment of this goal**.”*RJR*: “We believe that the issues raised by question 12 offer the IOM committee the unique opportunity to establish a framework for overseeing the design and execution of these types of studies and, ultimately, evaluating the data generated. Properly designing these groundbreaking studies will require the combined expertise of academia, the public health community, the government and industry.Key points to keep in mind: [paraphrasing] the design of any study in this arena will be largely product specific, the most meaningful studies will be done in the marketplace, significant differences may exist in what types of smokers are attracted to a particular new product, and [RJR] believe[s] that the most valuable type of research in this area will be a direct comparison of smokers' attitudes and perceptions before and after the new product introduction.”	“The committee makes the following recommendations:1. There is an urgent need for a national comprehensive surveillance system that collects information on a broad range of elements necessary to understand the population impact of tobacco products and PREPs, including attitudes, beliefs, product characteristics, product distribution and usage patterns, marketing messages such as harm reduction claims and advertising, the incidence of initiation and quitting and nontobacco risk factors for tobacco-related conditions. There should be surveillance of major smoking-related diseases as well as construction of aggregate population health measures of the net impact of conventional product and PREPs.2. The surveillance system should consist of mandatory, industry furnished data on tobacco product constituents, additives, and population distribution and sales.3. Resources should be made available for a program of epidemiological studies that specifically address the health outcomes of PREPs and conventional tobacco products, built on a robust surveillance system and using all available basic and clinical scientific findings.” (p. 197)

Unless otherwise indicated, emphasis is the authors'. PM, Philip Morris; RJR, R.J. Reynolds Tobacco.

The lawyers' influence is evident in PM's answer to a question about collaboration between industry, academia, and government, which was edited by Kevin Osborne, the in-house lawyer who reviewed PM's presentations to the IOM. The lawyers helped distinguish situations in which PM should openly disclose information to boost its credibility from situations in which such disclosure would reveal weaknesses or problems with the company's position. For example, in response to the IOM question about the “best mechanism to foster collaborative studies between tobacco industry, university, and other scientists” [102], PM wrote, “Philip Morris has already set up an ambitious program directed toward the development of harm-reduced products … [that] would involve Philip Morris support of external scientists through an independent funding mechanism” [110]. Osborne commented, “The ‘funding mechanism’ [the PM External Research Program] isn't independent; rather, the contemplated arrangement allows for the funding of independent research” [110]. The final version submitted to the IOM had no mention of PM's then-planned External Research Program.

**Figure 1:**
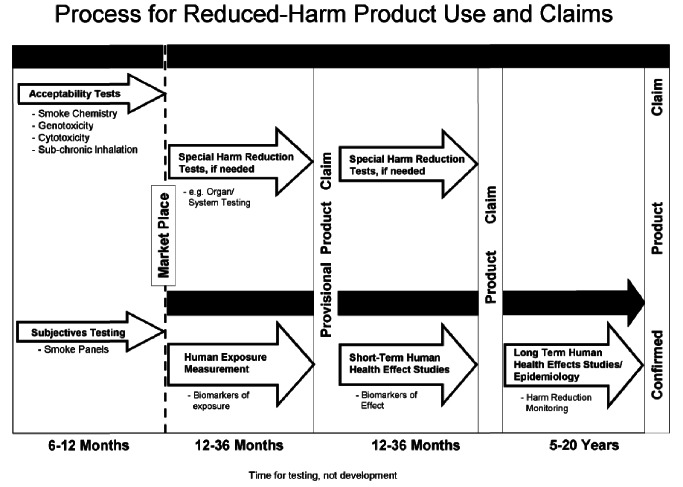
PM proposed timeline for assessment of reduced-harm products [219].

In response to the question about criteria for determining harm, PM recommended a tiered testing system that proposed as many as 20 years of surveillance to either confirm or invalidate a reduced harm claim (Figure 1). PM recommended that the validation process be divided between the premarket phase, with standard toxicology tests “to insure that a new product design change does not increase overall smoke chemistry or measured biological activity” [97] (called “acceptability,” referring to a specific level of acceptable harm and not to be confused with “consumer acceptability,” referring to consumer tastes and preferences), and post-market surveillance. The primary assessment of new products would take place largely in the post-market phase, because “due to the need for large numbers of smokers who currently use a product as their brand, it would be best to conduct the study in an after-market environment” [111]. Adopters of products with potential reduced-harms claims would serve as the test population and tobacco companies would benefit from rapid introduction of their products into the market.

Although the wording was changed slightly, Regulatory Principle 4 of the IOM report was similar to the tiered testing system PM recommended to the working group. Indeed, an internal draft statement prepared by PM WSA noted that IOM had accepted PM's model in all but name [112]. The WSA scientists proposed this schematic, and after a similar version was published in the IOM report, conducted a post hoc analysis that it would allow PM to make early health claims soon after smoke chemistry, toxicity, and the first round of human biomarker testing, prior to marketing [112],[113], and continue after-market testing among consumers for several years [101].

The IOM took a much firmer stance than the original PM proposal, setting high preliminary testing hurdles. Nevertheless, PM was pleased by the IOM's overall approach and continued to promote this tiered system to other organizations, including the WHO Scientific Advisory Committee on Tobacco Product Regulation later that year [114].

Among members of the academic and public health communities that were invited to present to the committee as independent experts, John Slade, a physician specializing in addiction and tobacco control, specifically tried to counter the influence of the tobacco companies on the IOM. In a presentation about the history of light and filtered cigarettes [115], Slade explained how tobacco companies and PM in particular generated controversy to confuse the public about the dangers of smoking. Since all documents presented to the IOM, including copies of presentations and written submissions, were publicly available, Slade obtained the submissions by the tobacco companies and sent letters to the committee reminding them that tobacco companies had a history of bad behavior and presented a point-by-point rebuttal to the companies' responses to the 12 questions [116],[117],[118]. Slade was also suspicious of RJR's claims for Eclipse and the industry's willing participation in establishing standards for risk reduced products, stating, “All three of the major cigarette manufacturers are eager to have reduced risk products on the market in their own terms, terms which guarantee the continuation of the public health fraud they have perpetuated for decades by promoting poisonous and addictive products to consumers of all ages” [117].

### Inviting IOM Committee Members to Apply for Funding from the Philip Morris External Research Program

A few months before the release of the IOM report, PM prepared to launch its new PM External Research Program (PMERP), nominally to fund independent research on harm reduction to aid product development [119],[120]. Solana sent identical letters to nine IOM committee members inviting them to apply for funding. Committee member, pathologist Adi Gazdar, planned to submit a research funding application. Prior to serving on the IOM committee, Gazdar had applied for and been refused money from the industry's Council for Tobacco Research [121],[122],[123],[124],[125],[126],[127],[128],[129],[130],[131],[132]. Kern Wildenthal, president of Gazdar's institution, University of Texas Southwestern at Dallas, prevented him from submitting the application. He described his reservations in a letter to Gazdar:

**[A]lthough Philip Morris intends to make use of peer review and to exert no control over publications after grants are awarded, they make it clear that peer review is only part of the picture and that the grant program is, in fact, for the benefit of the company and under the company's control.**
**Specifically, they state that “the purpose” of the program is to support “research that…enables Philip Morris to continue its pursuit of product modification(s) or new product design(s) that might reduce the health risk of smoking.” They also state that after peer review, Philip Morris has “final approval,” and that the company must be provided “assurances” that all studies they fund “serve relevant business needs” of the company. [emphasis original] [133]**

Wildenthal was correct to express reservations about PMERP. An independent retrospective analysis of the first round of funding of PMERP concluded that “the ostensible purpose of the programme is to help develop cigarette designs ‘that might reduce the health risk of smoking.’ Internal company documents also indicate that Philip Morris urgently seeks to restore its scientific ‘credibility,’ as part of a ‘new openness’ in relation to the external community” [120]. Gazdar's interest in PMERP nevertheless persisted. In 2003 he served as a member of PMERP's Scientific Advisory Board [134],[135],[136]. He also gave the keynote speech at the 2007 PMERP symposium, which showcased the work of scientists funded by the program [137].

Consistent with their desire to gain greater credibility in the scientific community, PM scientists shared information with or on behalf of committee members Henderson and Hatsukami at least once more after completion of the IOM report. Henderson invited the industry scientists to speak in a symposium she was organizing at a Society of Toxicology national meeting, on the “scientific basis of reducing harm from cigarette smoking” [138]. Hatsukami communicated with PM's Solana and Walk to obtain an overview of the company's research on biomarkers to assess reduced risk and exposure [139].

### The IOM Report Is Released

On February 22, 2001, the IOM released *Clearing the Smoke: Assessing the Science Base for Tobacco Harm Reduction* [3]. As part of the press event accompanying the report's release, Committee Chair Stuart Bondurant stated:

**The committee … concluded that the only way to ensure that the health claims made about these products are true, that the public is fully and accurately informed, and that the impact on the general population is positive is to use the potential capability of oversight, or regulation. We recommend that—in tandem with new surveillance and research efforts—regulatory principles [Box 1] be adopted to assure that the public is accurately informed about the health effects of new products, to prevent cigarettes with greater toxicity than those sold today from entering the market, and to gather complete information about new products…**
**We believe that manufacturers should have the necessary incentive to develop and market these products. What I mean by this is that there be a regulatory framework that is not so burdensome that manufacturers are not able to get these products to market but strict enough that they do in fact qualify as harm reduction products. [140]**

The report also concluded that harm reduction was feasible despite the fact that there had never been a potential reduced exposure product (PREP) that had been evaluated comprehensively enough to conclude that it actually reduced or would very likely reduce harm compared to conventional tobacco use. IOM recommended a research agenda that included describing the dose–response relationship between tobacco smoke/constituent exposure and health outcomes, surrogate markers of disease, preclinical research (describing but not specifically recommending the test battery proposed by PM, RJR, and B&W), and short- and long-term epidemiology and surveillance [3].

Internal PM emails among scientists, executives, and public relations staff reflected pleasure with the outcome [141],[142],[143],[144]. Ellen Merlo, Senior Vice President of Corporate Affairs, wrote, “If this is it, this is very good. Obviously, we agree and that's why we are working with public health officials and encouraging FDA regulation of the product… Very good positioning for us” [144]. Vice President of Federal Government Affairs John Scruggs agreed, “My initial view is that we should respond to the regulatory principles stated in the report *because they appear to track so well with our position* [emphasis added]” [145].

Mark Berlind, PM associate general counsel, emailed key company executives, communications staff, corporate affairs, and legal personnel to circulate a policy-oriented draft statement (it is unclear whether this was ever released to the public) reacting to the IOM report's 11 Regulatory Principles, noting that “there does seem to me to be … ways in which we could leverage them in both the FDA and WHO contexts”; both were organizations responsible for potential upcoming regulation [146].

PM gave a copy of the WRA draft statement to A&P to “see if anything in it is troubling as it relate[s] to legal/regulatory issues” [147]. Some copies of the draft statement are concealed behind attorney–client privilege claims [148],[149], but accessible versions permit comparisons. Earlier drafts contained more opinion and editorializing. For example, PM initially wrote that they considered the IOM's Regulatory Principles as an opportunity “in the spirit of continuing, constructive dialogue on these matters and in the hope of bringing diverse stakeholders together to find the common ground” [150], but removed this language from later drafts. Early drafts discussed how the Regulatory Principles could be applied to or whether they already existed in proposals for FDA regulatory legislation, WHO regulation, and the WHO Framework Convention on Tobacco Control; this discussion was also edited out for unspecified reasons. Finally, when addressing individual Regulatory Principles, PM initially accepted “nearly all” of them but opposed Principle 9, which stated that a regulatory agency should be empowered to set minimum performance standards for all tobacco products. Later drafts show that PM “accepted” all Regulatory Principles, including Principle 9 [150],[151],[152].

WSA began drafting an internal scientific response to support the statement being assembled by the lawyers and WRA. They identified statements and recommendations in the report and especially in the regulatory principles that supported PM's research priorities and positions on scientific issues. One WSA scientist wrote that Regulatory Principle 4 “seems to suggest that provided that the exposure reduction is shown to be large enough, exposure-reduction and risk-reduction claims can be made before clinical and epidemiological data are available [emphasis in original]” [113].

Lawyers also helped identify statements in the report that supported PM's research and business priorities. For example, PM lawyers Kevin Osborne and Paula Desel and A&P lawyer Rob Connelly discussed directions for evaluating the IOM report from scientific and policy standpoints with WSA's Solana [153].

PM's scientists considered Regulatory Principle 7, which said that manufacturers should be allowed to market conventional products (those without reduced exposure or reduced risk claims) without regulatory approval provided that they did not *increase* disease risk. The WSA's interpretation of the statement was that “We believe that our acceptability evaluations do this [prove that risk does not increase] for any new proposed product design changes” [112], meaning that no changes would occur in the evaluation of conventional tobacco products, despite the well-established evidence of their toxicity. The WSA's review of the IOM report was ultimately merged with the legal/regulatory statement written by the WRA [154], though it is unclear whether the final document was publicly released.

## Discussion

The tobacco industry has a history of producing and promoting misleading research to serve its business needs [1],[4],[5],[10],[11],[12],[13],[14],[15]. Consistent with this history, the tobacco companies used their legal and regulatory staff to access the IOM information-sharing process and used this access to deliver specific, carefully formulated messages to serve their business interests. They were satisfied with the results of the IOM report and devised ways to use the report's Regulatory Principles to accomplish their scientific and regulatory goals, some of which have continuing policy implications today.

The tobacco companies individually strategically organized to influence the IOM committee to win their favored scientific and regulatory recommendations. Using the advice of lawyers and consultants, PM initiated contact with the IOM committee by citing the precedent set by Canada's Expert Committees, which included industry scientists and stressed the importance of involving all stakeholders, as a reason why they should be asked to contribute their expertise to the IOM committee. Motivated to gather input from all stakeholders, the IOM committee invited PM and other tobacco companies to share information. This stance positioned the “health establishment” and the “tobacco industry” as two legitimate but polarized positions that needed to be skillfully mediated, a dynamic encouraged by the industry [155].

PM and RJR presentations [94],[97] (obtained from IOM public records) show that the companies denied the evidence that low-yield products harmed public health, stressed that they should be permitted to manufacture harm-reducing products that consumers would accept and buy [156], presented data from industry research which made cigarettes appear safer [93], and tried to secure protection to sell conventional cigarettes without additional restrictions [74],[111]. Limited scientific rationales for these positions were presented or, in the latter two cases, were nonexistent.

In contrast to the presumptions about openness, honesty, ethics, and neutrality, which are expected in scientific and academic discourse, the tobacco companies viewed their interactions with governing and regulatory bodies not as scientific and academic discourse but as an adversarial relationship to defend commercial interests. The presentations of the industry scientists were closely supervised by executives, regulatory specialists (PM's WRA), internal and external lawyers (A&P), and consultants (MBS' Jim Tozzi). (Many of the documents from PM, RJR, and B&W pertaining to the IOM are not accessible because the companies withheld them claiming attorney–client privilege [157],[158],[159],[160],[161],[162],[163],[164],[165],[166],[167],[168],[169],[170],[171],[172],[173],[174],[175],[176],[177],[178],[179],[180],[181],[182],[183],[184],[185],[186],[187],[188],[189],[190],[191],[192],[193]).

The involvement of lawyers in managing the tobacco industry's positions on scientific issues is longstanding, dating back over half a century [1]. In 2006, in response to a case brought by the US Department of Justice against the major cigarette companies [194], their lobbying arm (the Tobacco Institute), and their extramural research arms (including the Council for Tobacco Research) under the Racketeer-Influenced and Corrupt Organizations (RICO) Act, federal judge Gladys Kessler found that the tobacco companies had formed an illegal “enterprise” that engaged in “a massive 50-year scheme to defraud the public, including consumers of cigarettes, in violation of [the Racketeer Influenced and Corrupt Organizations (RICO) Act],” and that such behavior was continuing and likely to continue in the future [4]. Judge Kessler specifically highlighted the role of the lawyers in managing the tobacco industry's scientific efforts:

**At every stage, lawyers played an absolutely central role in the creation and perpetuation of the [racketeering] Enterprise and the implementation of its fraudulent schemes. They devised and coordinated both national and international strategy; they directed scientists as to what research they should and should not undertake; they vetted scientific research papers and reports as well as public relations materials to ensure that the interests of the Enterprise would be protected; they identified “friendly” scientific witnesses, subsidized them with grants from the Center for Tobacco Research and the Center for Indoor Air Research, paid them enormous fees, and often hid the relationship between those witnesses and the industry; and they devised and carried out document destruction policies and took shelter behind baseless assertions of the attorney client privilege. [4]**

### The Regulatory Principles in Action

Philip Morris used the IOM report's Regulatory Principles in its efforts to shape scientific standards and tobacco regulatory policy by quoting, and at times misinterpreting, these Regulatory Principles.

### Regulatory Principles 4 and 6: Substantial reduction in exposure/risk and post-market epidemiological surveillance

Regulatory Principles 4 and 6 (Box 1) allowed manufacturers to market tobacco products with exposure or risk reduction claims, provided that the products “substantially reduced” exposure as judged by independent scientific experts, and empowered a hypothetical regulatory agency to require manufacturers to conduct post-marketing surveillance and epidemiological studies to determine behavioral and health consequences of their products. Nominally following IOM regulatory Principle 4, PM contracted with the “independent” Life Sciences Research Office (LSRO) to develop criteria and a surveillance plan called the “Reduced Risk Review” that PM could then apply to its products. Far from being “independent,” PM was involved in selecting members of LSRO committees [195],[196]; 44% of the committee members assembled for the Reduced Risk Review had documented financial ties to the tobacco industry, with several working for tobacco companies PM, RJR, Liggett, Lorillard, Star Scientific, or Japan Tobacco ([196]; Table 1 identifies committee members with documented ties to the tobacco industry).

A year and a half after the IOM report was released and at the beginning of the LSRO project, Ed Carmines, a PM associate principal scientist, emailed Robin Philips, a PM business planning R&D engineer, to explain how PM was going to use LSRO in the context of the Regulatory Principles. With respect to Regulatory Principle number 4:

***LSRO is envisioned to represent PM's independent scientific experts to support the claims. The IOM did not identify what would be required only that it should undergo an independent review. After LSRO develops the criteria, we plan to ask them to review our data to see if it meets the criteria*…**
**The second relevant principle is number 6: It says the [*sic*] there should be a surveillance plan before the product is marketed to permit continuing review of the marketing claims. We want LSRO to identify what would be necessary in a surveillance plan and then to determine if our plans meet the pre-established criteria. [emphasis added] [197].**

LSRO assembled a committee for “Evaluating the Scientific Evidence for Potential Reduced-Risk Tobacco Products” that ultimately produced four monographs on scientific methods, biological effects assessment, exposure assessment, and differentiating the health risks of categories of tobacco products [198],[199],[200],[201]. The monographs recommended the same testing battery, analyses, and biomarkers, reference cigarettes, and machine testing protocols that PM and the other tobacco companies recommended to the IOM and that the IOM described in its report ([3], p. 292–293; [97],[111]).

It is important to emphasize that the second sentence in Regulatory Principle 4 states, “The *‘substantial reduction’ in exposure should be sufficiently large that measurable reduction in morbidity and/or mortality* (in subsequent clinical or epidemiological studies) *would be anticipated* as judged by independent scientific experts [emphasis added]” in order to preclude perhaps biologically irrelevant reductions in toxin exposures to form the basis for marketing claims to the public.

### Regulatory Principle 5: Claims must not be false or misleading

In 2001, as PM closely followed Congress's efforts to grant FDA jurisdiction over tobacco [202], key lawyers and executives at PM maintained a spreadsheet comparing elements of each proposed bill to standards in the IOM report [203],[204]. An undated position paper from the office of PM Vice President of Federal Government Affairs John Scruggs titled “Reduced Risk Tobacco Products: Full Disclosure vs. Government Suppression of Truthful and Non-Misleading Information” argued that the proposed bills, specifically one by Senator Ted Kennedy (D, MA), “appear[ed] to grant FDA authority to suppress information about reduced-risk or reduced-exposure tobacco products even if FDA has verified that these products, as a matter of science, genuinely have the potential to present reduced risks to individual consumers” [205]. The actual criteria set forth by the Kennedy Bill required that reduced risk products must not only reduce harm to individuals but also be otherwise appropriate to protect public health on a population level. PM did not agree with the latter criteria for withholding information about reduced risk claims and constructed a legal argument based on the First Amendment as well as statements made in the IOM report to argue that FDA should not have discretion to withhold information about verified claims:

**Proposals to give FDA regulatory authority over tobacco products take different approaches to so-called “reduced-risk” products. But once FDA makes a scientific determination about a particular product, neither the agency nor any other government body is Constitutionally permitted to suppress *truthful and non-misleading information about the product* [referring to *Clearing the Smoke* Regulatory Principle #5]. [emphasis in original] [205].**

The memo then quoted the IOM report, saying, “IOM added that the ‘*regulatory process should not discourage or impede scientifically grounded claims of reduced exposure, so long as steps are taken to ensure that consumers are not misled*…’ [emphasis added by PM author]” [205]. In short, PM considered using the IOM's regulatory principles (together with other legal arguments) to try to shape legislation that would maximize the company's ability to market FDA-recognized claims of reduced risk products using low standards.

While PM did not issue any statements publicly opposing the Kennedy bill using these legal arguments, what they did do was vigorously support a competing bill that was soundly opposed by the health groups. In 2001, PM supported alternative bills for tobacco regulation proposed by Representative Thomas M. Davis III (R-11^th^) and Senator Bill Frist (R-Tennessee) [17]. According to an independent analysis of tobacco documents related to regulation, PM's support of this legislation was based on the notion that “government regulation was part of PM USA's larger plan to be regarded as a normal, legitimate corporation, thereby ending its isolation and assuring its continued success” [17]. Public health groups were critical of the Davis and Frist bills. The Davis bill was declared to be “written on behalf of tobacco giant Philip Morris” in a position paper by the American Cancer Society, American Heart Association, American Lung Association, and Tobacco-Free Kids:

**In many critical sections, the bill changes the standard under which FDA normally operates from one that places concern for public health as the top priority to one that protects tobacco industry interests. Virtually every important section has a loophole or a standard that would prevent FDA from protecting public health. [206].**

Later that year, out of concern for the Davis and Frist bills “spinning out of control,” PM's Scruggs “concluded that Democratic Senator Edward Kennedy of Massachusetts was the key to success, and recommended negotiating with him to try to reach a compromise bill” [17], thus explaining why PM did not appear to publicly oppose the Kennedy bill.

### Regulatory Principle 7: Approval of products that do not claim to reduce risk or exposure

In their presentations and letters to the IOM, PM described a criterion of “acceptability,” defined as a product characteristic by which chemical or biological activity is no higher than “standard” [97],[111]. This “standard” was not defined but was implied to be conventional cigarettes currently on the market. For example, PM's response to the IOM's question about criteria to assert that a specific form of tobacco or tobacco product is less harmful than others stated, “We believe that as long as a new product does not increase the hazard of smoking, it should be allowed into commercial sales” [111]. The IOM report's Regulatory Principle 7 appeared to embody PM's principle of “acceptability”:

**In the absence of any claim of reduced exposure or reduced risk, manufacturers of tobacco products should be permitted to market new products or modify existing products without prior approval of the regulatory agency after informing the agency of the composition of the product and certifying that the product could not reasonably be expected to *increase* the risk of cancer, heart disease, pulmonary disease, adverse reproductive effects, or other adverse health effects, compared to similar conventional tobacco products, as judged on the basis of the most current toxicological and epidemiological information. ([3], p. 10).**

The 2009 Family Smoking Prevention and Tobacco Control Act (FSPTCA) [207] partially implemented Regulatory Principle 7's approach to new products by allowing manufacturers until March 22, 2011 to submit a report to the FDA if such products were “substantially equivalent” to products on the market on or before February 15, 2007, which would allow these products to be marketed unless the FDA acts to prohibit their sale (placing the burden on the FDA to disprove substantial equivalence) [208]. After March 22, 2011, products for which substantial equivalence is claimed may not be marketed until the agency acts. As of June 22, 2012, tobacco companies had submitted 3,303 applications for substantially equivalent products, 10 for modified risk tobacco products, and none for new tobacco products [209]; as of November 5, 2012, the FDA had not acted on any of these applications.

### The Need for Explicit Skepticism of Industry Scientific Claims

The extent of the industry's deception of the public and the scientific community and the role of industry lawyers in managing its scientific enterprise [1],[210],[211],[212] has become more apparent in the years since *Clearing the Smoke* was released. In addition to Judge Kessler's 2006 ruling that the major cigarette companies had formed a continuing illegal “enterprise” that was likely to continue in the future [4], the companies' manipulative behavior was also recognized in Article 5.3 of the WHO Framework Convention on Tobacco Control (FCTC), a treaty ratified by 176 parties as of July 2012, which requires that, “In setting and implementing their public health policies with respect to tobacco control, Parties shall act to protect these policies from commercial and other vested interests of the tobacco industry in accordance with national law” [213]. (The US has signed but not ratified the FCTC.)

In 2012, the IOM issued another FDA-commissioned report on regulation of tobacco products, *Scientific Standards for Studies on Modified Risk Tobacco Products* [214], which recommended minimum standards for scientific studies to support the marketing of modified-risk tobacco products and for postmarket studies of approved products. Similar to *Clearing the Smoke*, tobacco companies were permitted to make presentations to the new committee on study standards and study design and promoted similar ideas as in 2001, including the tiered testing system and consumer acceptability. The 2012 IOM report contextualized these industry contributions with an extensive discussion of the history of industry-funded research, the findings of the RICO lawsuit, and the danger posed to the FDA's reputation if it were to accept tobacco company–based research. In contrast to *Clearing the Smoke*, which only briefly mentioned but did not specifically recommend a minimal battery of tests, the new report conducted a thorough assessment of scientific studies including those mentioned in *Clearing the Smoke* in 2001 as well as the advances since then. In some ways, *Scientific Standards for Studies on Modified Risk Tobacco Products* improved on *Clearing the Smoke* by acknowledging that the tobacco industry has a well-documented record of scientific deception. However, even the IOM committee that prepared the 2011 report seemed to fail to take its own advice by permitting tobacco company scientists to present information to them. Any information submitted by tobacco interests should be treated with a high degree of skepticism in light of their history of deception documented by, among other things, the federal courts. While the US has not yet ratified the FCTC, and the FSPTCA requires nonvoting representatives on the FDA's Tobacco Products Scientific Advisory Committee [215], the FDA and advisory bodies such as the IOM should implement the guidelines for Article 5.3 “to protect [public health] policies from commercial and other vested interests of the tobacco industry in accordance with national law” [213]. While it may not be possible to exclude tobacco interests from presenting information to independent and government scientific and regulatory decision-making bodies, these bodies should be mindful of Principle 1 of the Guiding Principles for implementation of Article 5.3 of the FCTC which states, “There is a fundamental and irreconcilable conflict between the tobacco industry's interests and public health policy interests” [216].

### Policy Implications

Many tobacco company ideas appeared in the final IOM report, and some have policy implications that were continuing to reverberate in 2012. The main ideas promoted by the tobacco industry to the IOM, as described in the Results section, were: (1) ability to market and sell potential reduced exposure products that pass initial acceptability tests, and to continue selling them for years in order to conduct epidemiological surveillance for health effects (a tiered testing system) [97],[101],[111],[112], (2) that harm reduction should be considered relative to conventional cigarettes, as opposed to absolute harm from baseline [97],[111],[112], (3) a mechanism to sell “substantially equivalent” products that were *not more harmful* (but not necessarily less harmful) than existing products [97],[111], and (4) the notion that reduced-harm products must appeal to consumers in order to be marketable and effective [94],[97].

An inherent limitation in the whole process that led to *Clearing the Smoke* was that the FDA was looking for assistance as to standards it should apply to tobacco company applications to market cigarette-like products (like RJR's Eclipse) that would be less harmful than cigarettes. The FDA's and IOM's assumption was that there might indeed be such products and that allowing companies to market them with proper restrictions might improve the public's health. Hence, ideas like making PREPs unattractive to consumers or banning them if they were more dangerous than not smoking at all were simply off the table.

The IOM's stance on consumer acceptability—which was consistent with the tobacco companies' imperative to sell their products—overlooks the regulatory option of reducing tobacco use by requiring that products be less “acceptable” by prohibiting the use of additives (such as menthol [155],[217]) that make the cigarettes less harsh and easier to smoke. In a well-regulated market that imposes high barriers on products that aim to please the consumer, less-acceptable products could be effective at reducing smoking-related disease.

The major regulatory goal that the industry aimed for but did not achieve was to restrict the evaluation of risk or exposure reduction to the individual level only. An analysis of the tobacco industry documents by McDaniel and Malone found that, “PM want[ed] reduced risk tobacco products to be regulated by the FDA, but it did not support applying a public health standard to such products. A public health standard would require the FDA to withhold approval from reduced risk cigarettes if they led to an increase in the incidence of smoking among the population by causing fewer people to quit or causing quitters to resume smoking. Instead, PM preferred a standard that focused on the benefits of reduced risk products for individual adult smokers” [17]. Consistent with this position, both PM and RJR answered the IOM's questions about population-level studies with nonspecific, vague responses (Table 1). Later PM objected to the proposed Kennedy bill for tobacco regulation, because it required a public health standard as well as an individual standard of harm reduction and empowered the FDA to withhold information about tobacco products if they did not meet both criteria. Despite the industry's urging, the IOM concluded that reduced-harm products must protect at both the individual and population levels and devoted a chapter of the report to population-based surveillance. Attention to population-level effects is important, because it is possible that a product that represented lower risk to an individual (such as smokeless tobacco compared to smoking cigarettes) could still increase population-level harm if the net effect was to reduce cessation of all tobacco use and promote initiation (even of the lower risk product) or dual use of the new product and cigarettes by the same people [218]. (Indeed, after the major US cigarette companies purchased smokeless tobacco companies they started promoting dual use of co-branded snus (oral, smokeless tobacco) and cigarettes with snus used in smoke-free environments such as bars and airplanes, and cigarettes at other times [218].) The FSPTCA also includes a provision requiring that modified-risk tobacco products can only be defined as such if they reduce harm for individuals *and* the population.

### Limitations

As a result of the 1998 Minnesota settlement, the 1998 Master Settlement Agreement, and the federal RICO ruling, tobacco companies are required to make internal documents produced in discovery in smoking and health litigation publicly available. As a result, the Legacy Tobacco Documents Library provides important insights into the tobacco industry's approach to the IOM Committee to Assess the Science Base for Tobacco Harm Reduction, but some key documents remain withheld by tobacco companies (under privilege and confidentiality claims that apply to documents being used in anticipation of litigation or related to trade secrets and personal information) including drafts of presentations from PM, RJR, and B&W to the IOM working group [157],[158],[159],[160],[161],[162],[163],[164],[165],[166],[167],[168],[169],[170],[171],[172],[173],[174],[175],[176],[177],[178],[179],[180],[181],[182],[183],[184],[185],[186],[187],[188],[190],[191],[192] and drafts of PM's reaction to the report [148],[149],[189],[193]. Despite this limitation, the available documents reveal extensive coordination among industry scientists, lawyers, executives, and regulatory staff in the presentation of information to the IOM toward the fulfillment of the industry's scientific, regulatory, and business goals.

The written record alone in the form of available industry and IOM documents does not provide sufficient evidence to claim cause and effect, i.e. that the scientific information presented by the companies yielded a particular outcome. However, the evidence does show that the industry had certain goals they wanted to accomplish and were generally pleased with and able to leverage the *Clearing the Smoke* report to promote their business agendas.

## Conclusion

The IOM report *Clearing the Smoke* was an early attempt to grapple with the complex scientific and regulatory issues surrounding the possibility of reduced-harm tobacco products. The relative lack of information in the field at the time created a void that the tobacco industry sought to fill with its own data and ideas about how reduced-harm products should be evaluated, regulated, and sold. The IOM committee's mandate was predicated on the belief of some in the public health community that lives could be saved if such a consumer-acceptable reduced-harm tobacco product was available. At the same time, it was understood then—and understood even better now—that many safeguards would be necessary to make sure that the product was not misrepresented as being safe when it was not (like filtered cigarettes and “low tar” cigarettes) and that it did not cause more harm by persuading people that it was safe to start or not to quit. While *Clearing the Smoke* states, “The committee believes that harm reduction is feasible and justified public health policy—but only if it is implemented carefully” and that “The effect of PREPS could be to increase or decrease tobacco-related disease in the population” [3], others in the public health community are very skeptical about such products. Readers should not be left with the impression that the fact that this skeptical point of view did not prevail is necessarily suggestive of tobacco industry influence. Rather, they should be aware of the complex strategies that tobacco companies have been using to attempt to influence regulatory policy and the urgent need to protect future endeavors.

There was a lack of clear policy on tobacco industry engagement by the IOM which, combined with the general presumption of honesty upon which all scientific discourse is based, created an opportunity for the tobacco companies to advocate positions that supported their interests. The industry took advantage of this situation and, in the end, some of the industry recommendations were reflected in *Clearing the Smoke* and the subsequent legislation assigning the FDA regulatory authority over tobacco products. The presence of tobacco industry representatives on the FDA's Tobacco Products Scientific Advisory Committee [215], combined with the FDA's official consideration of the tobacco industry as a “stakeholder,” increase the likelihood that the tobacco companies will continue to successfully manipulate the scientific discourse around tobacco product regulation, to the companies' benefit and to the detriment of public health. To prevent such an outcome, the FDA and counterpart organizations in other countries need to remain cognizant of the guidelines for implementing FCTC Article 5.3 [213] and that they are dealing with companies with a history of more than 50 years of intentionally misleading the public and who were found by two federal courts to have participated in “a pattern of racketeering activity” in violation of the RICO Act [4] when assessing the role of the tobacco companies and the information they present as part of the regulatory process.

## Supporting Information

Alternative Language Abstract S1 German translation of the abstract.
(DOCX)

## Abbreviations

A&P: Arnold & Porter
B&W: Brown & Williamson
FDA: US Food and Drug Administration
FCTC: WHO Framework Convention on Tobacco Control
FSPTCA: Family Smoking Prevention and Tobacco Control Act
INBIFO: Institut für Biologische Forschung
IOM: Institute of Medicine
LSRO: Life Sciences Research Office
LTDL: UCSF Legacy Tobacco Documents Library
MBS: Multinational Business Services, Inc
NAE: National Academy of Engineering
NAS: National Academy of Sciences
NRC: National Research Council
PARO: Public Access Records Office
PM: Philip Morris
PMERP: PM External Research Program
PREP: potential reduced exposure product
RICO: Racketeer Influenced and Corrupt Organizations
RJR: R.J. Reynolds Tobacco
WHO: World Health Organization
WRA: Philip Morris Worldwide Regulatory Affairs division
WSA: Philip Morris Worldwide Scientific Affairs division
