# Supplementary material for: Tobacco Company Efforts to Influence the Food and Drug Administration-Commissioned Institute of Medicine Report Clearing the Smoke: An Analysis of Documents Released through Litigation
Source: PLoS Med. 2013 May 28;10(5):e1001450. doi: 10.1371/journal.pmed.1001450 (PMC3665841; doi:10.1371/journal.pmed.1001450)
Supplement: Alternative Language Abstract S1 — German translation of the abstract. (DOCX) [file pmed.1001450.s001.docx]

**DEUTSCHES ABSTRAKT**

**Hintergrund:** Motiviert von der Schaffung schadensreduzierter Tabakprodukte, beauftragte die US Food and Drug Administration (FDA) das US Institute of Medicine (IOM), wissenschaftliche Grundlagen für weniger schädliche Tabakprodukte zu evaluieren. Dies führte zum IOM-Report „*Clearing the Smoke“, der* 2001 erschien*.*

**Fragestellung:** Untersucht wurde, wie sich die Tabakhersteller organisierten, um das für den Report verantwortliche IOM Komitee zu beeinflussen.

**Methode und Ergebnisse:** Vormals geheime Tabakindustriedokumente und öffentlich zugängliche Unterlagen des IOM wurden in der Legacy Tobacco Documents Library der University of California, San Francisco ausgewertet. (Methodische Limitationen ergeben sich aus dem Umstand, dass möglicherweise relevante Dokumente von der Tabakindustrie nicht zugänglich gemacht wurden). Die Tabakhersteller maßen dem IOM Report hohe regulatorische Wirkung zu. Gemeinsam mit Unternehmensberatern und Anwaltskanzleien entwickelten sie Strategien, um Zugang zum Verfahren des IOM zu erlangen. Als Studienverantwortliche des IOM die Firmen einluden, ihre Erkenntnisse über Exposition und Erkrankung, klinische Studiendesigns für Sicherheit und Wirksamkeit sowie Auswirkungen auf Rauchbeginn und Abstinenz vorzustellen, gestalteten Anwälte und Berater der Tabakhersteller sowie firmeneigene Experten für Regulation gemeinsam mit Wissenschaftlern der Tabakindustrie die Präsentationen. Obwohl die zur Verfügung stehenden Belege nicht ausreichen, um Ursache-Wirkungs-Beziehungen zu beweisen, und das IOM auch ohne den Einfluss der Tabakindustrie zu denselben Schlüssen hätte gelangen können, waren die Hersteller mit dem endgültigen Bericht zufrieden. Die besondere Zustimmung der Industrie fanden Empfehlungen für ein gestaffeltes Zulassungssystem (mit gesonderten Stufen für Exposition und Risiko, wovon man sich Erleichterungen für die Zulassung versprach) und die Erlaubnis Produkte, die konventionellen Zigaretten vergleichbar sind („substanzielle Äquivalenz“) ohne vorrausgehende Zustimmung der Regulierungsbehörde in den Handel zu bringen. Einige Grundsätze des IOM-Reports wurden in den Family Smoking Prevention and Tobacco Control Act (US-Gesetz zur Prävention des Rauchens in Familien und zur Bekämpfung des Tabakkonsums) von 2009 übernommen.

**Schlussfolgerung:** Die Tabakhersteller interagierten strategisch mit dem IOM, um verschiedene für sie günstige wissenschaftliche und regulatorische Empfehlungen zu erlangen.
